# Supplementary material for: Optimising Seniors’ Metabolism of Medications and Avoiding Adverse Drug Events Using Data on How Metabolism by Their P450 Enzymes Varies with Ancestry and Drug–Drug and Drug–Drug–Gene Interactions
Source: J Pers Med. 2020 Aug 11;10(3):84. doi: 10.3390/jpm10030084 (PMC7563167; doi:10.3390/jpm10030084)
Supplement: Supplementary file 1 [file jpm-10-00084-s001.pdf]

Table S1. Gene-drug pairs and dose recommendations by the Clinical Pharmacogenetics Implementation Consortium (CPIC) and the Dutch Pharmacogenetics Working Group (DPWG)[8]

Drug	Gene-drug Interaction, Phenotype, Therapeutic Recommendations and [Classifications of Evidence]	Ref
**P450 Cytochrome Isoform CYP2C9**		
Phenytoin	CPIC: Consider for IM 25% [M] and for PM 50% [S] reduction of recommended starting maintenance dose. Subsequent doses should be adjusted according to therapeutic drug monitoring and response.	9
	DPWG: Standard loading dose. For IM 25% [4D] and for PM 50-60% [4D] reduction in maintenance dose. Evaluate response and serum concentration after 7–10 days. Be alert to ADEs (e.g., ataxia, nystagmus, dysarthria, sedation).	10-12
Warfarin	CPIC: For all these allele combinations *1/*2 *1/*3 *2/*2 *2/*3 *3/*3 Calculate dose based on validated published pharmacogenetic algorithm [S].	13,14
	DPWG: *1/*2 Initiate therapy with recommended starting dose [4A].<br>*1/*3 [4D] and *2/*2 [4A] Consider a reduction to 65% of the normal starting dose.<br>*2/*3 [4A] and *3/*3 [4C] Consider a reduction to 45% of the normal starting dose.	12
**P450 Cytochrome Isoform CYP2C19**		
Amitriptyline	CPIC: IM Initiate therapy with recommended starting dose [S].<br>PM (1) Avoid amitriptyline use due to potential for sub-optimal response. Consider alternative drug not metabolized by CYP2C19. TCAs without major CYP2C19 metabolism include nortriptyline and desipramine. (2) Consider 50% reduction of recommended starting dose. Utilize therapeutic drug monitoring to guide dose adjustments [M].<br>UM or RM: (1) Avoid amitriptyline use due to potential for sub-optimal response. Consider alternative drug not metabolized by CYP2C19. TCAs without major CYP2C19 metabolism include nortriptyline and desipramine. (2) If amitriptyline is warranted, utilize therapeutic drug monitoring to guide dose adjustments [O].	15,16
	DPWG: IM, PM, UM Initiate therapy with recommended starting dose [IN].	12
Citalopram/ Escitalopram	CPIC: IM Initiate therapy with recommended starting dose [S].<br>PM (1) Consider a 50% reduction of recommended starting dose and titrate to response. (2) Select alternative drug not predominantly metabolized by CYP2C19 [M].<br>UM Consider an alternative drug not predominantly metabolized by CYP2C19 [M].	17

	DPWG: IM (1) Consider a maximum daily dose of 20 mg for age < 65 or 10 mg for ≥ 65 years. (2) Consider a 50% reduction in starting dose and raise to normal dose under monitoring of ECG to 40 mg for age < 65 or 20 mg for ≥ 65 years [4A].<br>PM Consider a maximum daily dose of 20 mg for age < 65 or 10 mg for ≥ 65 years [4A].<br>UM Initiate therapy with recommended starting dose [3AA].	11,12
Clopidogrel	CPIC: IM [M], PM [S] Consider alternative drug not metabolized by CYP2C19.<br>UM Initiate therapy with recommended starting dose [S].	18,19
	DPWG: IM, PM Consider alternative drug not metabolized by CYP2C19 [4F].<br>UM Initiate therapy with recommended starting dose [4A].	11,12
Clomipramine	CPIC: IM Initiate therapy with recommended starting dose [O].<br>PM (1) Avoid clomipramine use due to potential for sub-optimal response. Consider alternative drug not metabolized by CYP2C19. TCAs without major CYP2C19 metabolism nortriptyline and desipramine, (2) Consider 50% reduction of recommended starting dose. Utilize therapeutic drug monitoring to include guide dose adjustments [O].<br>UM or RM (1) Avoid clomipramine use due to potential for sub-optimal response. Consider alternative drug not metabolized by CYP2C19. TCAs without major CYP2C19 metabolism include nortriptyline and desipramine. (2) If clomipramine is warranted, utilize therapeutic drug monitoring to guide dose adjustments [O].	15,16
	DPWG: IM, PM, UM Initiate therapy with recommended starting dose.	12
Doxepin	CPIC: IM Initiate therapy with recommended starting dose [O].<br>PM (1) Avoid doxepin use due to potential for sub-optimal response. Consider alternative drug not metabolized by CYP2C19. TCAs without major CYP2C19 metabolism include nortriptyline and desipramine. (2) Consider 50% reduction of recommended starting dose. Utilize therapeutic drug monitoring to guide dose adjustments [O].<br>UM or RM (1) Avoid doxepin use due to potential for sub-optimal response. Consider alternative drug not metabolized by CYP2C19. TCAs without major CYP2C19 metabolism include nortriptyline and desipramine. (2) If doxepin is warranted, utilize therapeutic drug monitoring to guide dose adjustments [O].	015,16
	DPWG: IM, PM, UM Initiate therapy with recommended starting dose [IN].	12
Imipramine	CPIC: IM Initiate therapy with recommended starting dose [O].	15,16

	PM (1) Avoid imipramine use due to potential for sub-optimal response. Consider alternative drug not metabolized by CYP2C19. TCAs without major CYP2C19 metabolism include nortriptyline and desipramine. (2) Consider 50% reduction of recommended starting dose. Utilize therapeutic drug monitoring to guide dose adjustments [O].<br>UM or RM (1) Avoid imipramine use due to potential for sub-optimal response. Consider alternative drug not metabolized by CYP2C19. TCAs without major CYP2C19 metabolism include nortriptyline and desipramine. 2) If imipramine is warranted, utilize therapeutic drug monitoring to guide dose adjustments [O].	
	DPWG: IM Initiate therapy with recommended starting dose [4A].<br>PM (1) Consider a 30% reduction of recommended starting dose and utilize therapeutic drug monitoring of imipramine and desipramine. 2) Consider alternative drug not metabolized by CYP2C19 [4A].<br>UM Initiate therapy with recommended starting dose [4A].	11,12
Sertraline	CPIC: IM Initiate therapy with recommended starting dose [S].<br>PM (1) Consider a 50% reduction of recommended starting dose and titrate to response. (2) Select alternative drug not predominantly metabolized by CYP2C19 [O].<br>UM: Initiate therapy with recommended starting dose. If patient does not respond to recommended maintenance dosing, consider alternative drug not predominantly metabolized by CYP2C19 [O].	17
	DPWG: IM Consider a maximum daily dose of 100 mg and utilize clinical monitoring on response/side effects or therapeutic drug monitoring of sertraline + desmethylsertraline to guide dose adjustments [4A].<br>PM Consider a maximum daily dose of 50 mg and utilize clinical monitoring on response/side effects or therapeutic drug monitoring of sertraline + desmethylsertraline to guide dose adjustments [4C].<br>UM: Initiate therapy with recommended starting dose [4C].	11,12
**P450 Cytochrome Isoform CYP2D6**		
Amitriptyline	CPIC: IM Consider 25% reduction of recommended starting dose. Utilize therapeutic drug monitoring to guide dose adjustments [M].<br>PM: (1) Avoid amitriptyline use due to potential for side effects. Consider alternative drug not metabolized by CYP2D6. (2) If amitriptyline is warranted, consider 50% reduction of recommended starting dose. Utilize therapeutic drug monitoring to guide dose adjustments [S].	17,18

	UM: (1) Avoid amitriptyline use due to potential lack of efficacy. Consider alternative drug not metabolized by CYP2D6. (2) If amitriptyline is warranted, consider titrating to a higher target dose (compared to normal metabolizers). Utilize therapeutic drug monitoring to guide dose adjustments [S].	
	DPWG: IM (1) Consider alternative drug not metabolized by CYP2D6. (2) If an alternative is not possible consider a decrease of up to 60% of the recommended dose under therapeutic drug monitoring of amitriptyline and nortriptyline [3C].<br>PM: (1) Consider alternative drug not metabolized by CYP2D6. (2) If an alternative is not possible consider a decrease of up to 50% of the recommended dose under therapeutic drug monitoring of amitriptyline and nortriptyline [3A].<br>UM: (1) Consider alternative drug not metabolized by CYP2D6. (2) If an alternative is not possible consider an increase of up to 125% of the recommended dose under therapeutic drug monitoring of amitriptyline and nortriptyline. Be alert of a possible decrease in therapeutic levels and an increase of active cardiotoxic hydroxymetabolites [3C].	11,12
Clomipramine	CPIC: IM Consider 25% reduction of recommended starting dose. Utilize therapeutic drug monitoring to guide dose adjustments [O].<br>PM: (1) Avoid clomipramine use due to potential for side effects. Consider alternative drug not metabolized by CYP2D6. (2) If clomipramine is warranted, consider 50% reduction of recommended starting dose. Utilize therapeutic drug monitoring to guide dose adjustments [O].<br>UM: (1) Avoid clomipramine use due to potential lack of efficacy. Consider alternative drug not metabolized by CYP2D6. (2) If clomipramine is warranted, consider titrating to a higher target dose (compared to normal metabolizers). Utilize therapeutic drug monitoring to guide dose adjustments [O].	15,16
	DPWG: IM (1) Consider 30% reduction of recommended starting dose. (2) Utilize therapeutic drug monitoring of clomipramine and desmethylclomipramine [4C].<br>PM: Depression: (1) Consider 60% reduction of recommended starting dose. (2) Utilize therapeutic drug monitoring of clomipramine and desmethylclomipramine. Anxiety: (1) Consider alternative drug not metabolized by CYP2D6. (2) If an alternative is not possible consider a decrease of up to 50% of the recommended dose under therapeutic drug monitoring of clomipramine and desmethylclomipramine. [4C].<br>UM: (1) Consider alternative drug not metabolized by CYP2D6. (2) If an alternative is not possible consider an increase of up to 150% of the recommended dose under therapeutic drug monitoring of amitriptyline and nortriptyline. Be alert of a possible decrease in therapeutic levels and an increase of cardiotoxic active hydroxymetabolites [3C].	10-12

Codeine	CPIC: IM Use label-recommended age- or weight-specific dosing. If no response, consider alternative analgesics such as morphine or a non-opioid [M].<br>PM: Avoid codeine use due to lack of efficacy [S].<br>UM: Avoid codeine use due to potential for toxicity [S].	20,21
	DPWG: IM Cough: no action required. Pain: Be on alert for a lack of clinical effect. In case of a lack of clinical effect consider a raise in daily dose or consider an alternative drug [3A].<br>PM: Cough: no action required. Pain: Consider an alternative drug [4B].<br>UM: Contraindicated [3F].	10-12
Doxepin	CPIC: IM Consider 25% reduction of recommended starting dose. Utilize therapeutic drug monitoring to guide dose adjustments [O].<br>PM: (1) Avoid doxepin use due to potential for side effects. Consider alternative drug not metabolized by CYP2D6. (2) If doxepin is warranted, consider 50% reduction of recommended starting dose. Utilize therapeutic drug monitoring to guide dose adjustments [O].<br>UM: (1) Avoid doxepin use due to potential lack of efficacy. Consider alternative drug not metabolized by CYP2D6. (2) If doxepin is warranted, consider titrating to a higher target dose (compared to normal metabolizers). Utilize therapeutic drug monitoring to guide dose adjustments [O].	15,16
	DPWG: IM: Consider a 20% reduction of recommended starting dose. Utilize therapeutic drug monitoring to monitor doxepin and nordoxepin to guide dose adjustments [3A].<br>PM: Consider a 60% reduction of recommended starting dose. Utilize therapeutic drug monitoring to monitor doxepin and nordoxepin to guide dose adjustments [3F].<br>UM: (1) Consider alternative drug not metabolized by CYP2D6. (2) If an alternative is not possible consider an increase of up to 200% of the recommended dose under therapeutic drug monitoring of doxepin and nordoxepin [3A].	11,12
Fluvoxamine	CPIC: IM: Initiate therapy with recommended starting dose [M].<br>PM: (1) Consider a 25–50% reduction of recommended starting dose and titrate to response. (2) Use an alternative drug not metabolized by CYP2D6 [O].<br>UM: No recommendation due to lack of evidence [O].	19
	DPWG: IM [IN], PM [3AA] UM [IN] Initiate therapy with recommended starting dose.	12
Imipramine	CPIC: IM: Consider 25% reduction of recommended starting dose. Utilize therapeutic drug monitoring to guide dose adjustments [O].	15,16

	PM: (1) Avoid imipramine use due to potential for side effects. Consider alternative drug not metabolized by CYP2D6. (2) If imipramine is warranted, consider 50% reduction of recommended starting dose. Utilize therapeutic drug monitoring to guide dose adjustments [O].<br>UM: (1) Avoid imipramine use due to potential lack of efficacy. Consider alternative drug not metabolized by CYP2D6. (2) If imipramine is warranted, consider titrating to a higher target dose (compared to normal metabolizers). Utilize therapeutic drug monitoring to guide dose adjustments [O].	
	DPWG: IM: Consider 30% reduction of recommended starting dose. Utilize therapeutic drug monitoring to guide dose adjustments [4A].<br>PM: Consider 70% reduction of recommended starting dose. Utilize therapeutic drug monitoring to guide dose adjustments [4C].<br>UM: (1) Consider alternative drug not metabolized by CYP2D6. (2) If an alternative is not possible consider an increase of up to 170% of the recommended dose under therapeutic drug monitoring of imipramine and desipramine [4A].	10-12
Nortriptyline	CPIC: IM Consider 25% reduction of recommended starting dose. Utilize therapeutic drug monitoring to guide dose adjustments [M].<br>PM: (1) Avoid nortriptyline use due to potential for side effects. Consider alternative drug not metabolized by CYP2D6. (2) If nortriptyline is warranted, consider 50% reduction of recommended starting dose. Utilize therapeutic drug monitoring to guide dose adjustments [S].<br>UM: (1) Avoid nortriptyline use due to potential lack of efficacy. Consider alternative drug not metabolized by CYP2D6. (2) If nortriptyline is warranted, consider titrating to a higher target dose (compared to normal metabolizers). Utilize therapeutic drug monitoring to guide dose adjustments [S].	15,16
	DPWG: IM: Consider a 40% reduction of recommended starting dose. Utilize therapeutic drug monitoring of nortriptyline and 10hydroxytriptyline to guide dose adjustments [4C].<br>PM: Consider a 60% reduction of recommended starting dose. Utilize therapeutic drug monitoring of nortriptyline and 10hydroxytriptyline to guide dose adjustments [3C].<br>UM: (1) Consider alternative drug not metabolized by CYP2D6 (2) If an alternative is not possible consider an increase of up to 60% of the recommended dose. Utilize therapeutic drug monitoring of nortriptyline and 10-hydroxytriptyline to guide dose adjustments [3C].	11,12
Paroxetine	CPIC: IM: Initiate therapy with recommended starting dose [M].<br>PM: (1) Consider alternative drug not predominantly metabolized by CYP2D6. (2) If paroxetine use warranted, consider a 50% reduction of recommended starting dose and titrate to response [O].<br>UM: Consider alternative drug not predominantly metabolized by CYP2D6 [S].	19
	DPWG: IM: Initiate therapy with recommended starting dose [4A].	10-12

	PM: Initiate therapy with recommended starting dose [4A].<br>UM: Consider alternative drug not metabolized by CYP2D6 [4C].	
**Gene SLCO1B1**		
Simvastatin	CPIC: Decreased Function (521TC). and Poor Function (521CC): (1) Prescribe a lower dose. (2) Consider an alternative statin (e.g., pravastatin or rosuvastatin). (3) Consider routine CK surveillance [S].	22,23
	DPWG: 521TC (1) Consider alternative drug. (2) If simvastatin is warranted, prescribe a maximum dose of 40 mg/day [4D].<br>521CC: Select alternative drug [4D].	12
**Gene VKORC1**		
Warfarin	CPIC: -1639GA Calculate dose based on validated published pharmacogenetic algorithm [S].<br>-1639AA Calculate dose based on validated published pharmacogenetic algorithm [S].	13,14
	DPWG: -1639GA Initiate therapy with recommended starting dose [4A].<br>-1639AA Consider a reduction to 60% of the normal starting dose [4A].	12

Source: Bank, P.C.D.; Caudle, K.E.; Swen, J.J.; Gammal, R.S.; Whirl-Carrillo, M.; Klein, T.E.; Relling, M.V.; Guchelaar, H-J. A comparison of the guidelines of the Clinical Pharmacogenetics Implementation Consortium and the Dutch Pharmacogenetics Working Group. Clin Pharmacol Ther. **2018,** *103(4),* 599–618.

Notes: 1* and similar notations denote alleles of enzymes; IM = intermediate metabolizer; NM = normal metabolizer; PM = poor metabolizer; RM = rapid metabolizer; UM = ultra-rapid metabolizer; ADE = adverse drug event; M = moderate; S = strong; O = Optional; IN = Insufficient evidence; 0 = data on file; 1 = published incomplete case reports; 2 = well documented case reports / case series; 3 = published controlled studies of moderate quality; 4 = published controlled studies of good quality; A = minor clinical effect; B = clinical effect : short-lived discomfort (<48 h) without permanent injury; C = clinical effect: long-standing discomfort (48–168 h) without permanent injury; D = clinical effect: long-standing effect (>168) and permanent symptom or invalidating injury; E = Increased risk of failure of lifesaving therapy / expected bone marrow depression; F = death, arrhythmia, unexpected bone marrow depression

Table S2. Effect of polymorphisms in drug-drug interactions

P450 cytochrome isoform CYP2C9

**Effector drug (dose)**	**Substrate (dose)**	**Genotype/phenotype†**	**Effect of interaction**		**Clinical impact of interaction**	**Possible mechanism**	**Level of evidence, citation**
			**Pharmacodynamics**	**Pharmacokinetics**			

NSAID	Coumarins	CYP2C9*1 (normal)<br>CYP2C9*2; CYP2C9*3 (decreased)	The risk of over-anti coagulation (INR ≥6) = 1.69 (95% CI: 1.05–02.69)<br>The risk of over-anti coagulation (INR ≥6) = 2.28 (95% CI: 1.06–04.90)	NA	Substantial<br>Major	Reduced capacity of CYP2C9; CYP2C9 inhibition	3,26
NSAID (standard daily dose)	Acenocoumarol (age dependent)	CYP2C9*1/*2; *1/*3 (IM)	(INR <4.9)	NA	Major		3,25
Simvastatin	Warfarin	CYP2C9*1/*3 (IM)<br>CYP2C9*2/*3 (PM)<br>CYP2C9*3/*3 (PM)		Dose ↓ 25%<br>Dose ↓ 25%<br>Dose ↓ 43%	Substantial<br>Substantial<br>Substantial	Inhibition of CYP2C9*3 dependent warfarin metabolism	3,27
Fluconazole (200 mg)	Flurbiprofen (50 mg)	CYP2C9*1/*1 (NM)<br>CYP2C9*1/*3 (IM)<br>CYP2C9*3/*3 (PM)	NA	AUC ↑ 102%<br>AUC ↑ 79%<br>AUC ↑ 41%	Substantial<br>Moderate<br>Moderate	CYP2C9 inhibition	3,28
Fluconazole (400 mg)	Flurbiprofen (50 mg)	CYP2C9*1/*1 (NM)<br>CYP2C9*1/*3 (IM)	NA	AUC ↑ 203%<br>AUC ↑ 148%	Substantial<br>Substantial	CYP2C9 inhibition	3,28
Valproic Na (1st week = 200 mg and	Losartan (25 mg)	CYP2C9*1/*1 (NM	NA	MR of losartan/E3174 = 1.8; Ratio = 2.7	Substantial<br>Substantial<br>Substantial	CYP2C9 inhibition	3,29

next 3 weeks = 400 mg)		CYP2C9*1/*2) (IM) CYP2C9*1/*3 (IM)		MR = 1.5; Ratio = 2.5 MR = 5.28; Ratio = 4.8			
Rifampicin (450 mg)	Tolbutamide (500 mg)	CYP2C9*1/*1 (NM) CYP2C9*1/*2 (IM) CYP2C9*1/*3 (IM) CYP2C9*2/*3 (PM) CYP2C9*3/*3 PM)	NA	Clearance ↑ 97% Clearance ↑ 84% Clearance ↑ 92% Clearance ↑ 70% Clearance ↑ 162%	Moderate Moderate Moderate Moderate Substantial	CYP2C9 induction	3,30

P450 Isoform CYP2C19

**Effector drug (dose)**	**Substrate (dose)**	**Genotype/ phenotype†**	**Effect of interaction**		**Clinical impact of interaction**	**Possible mechanism**	**Level of evidence [citation]**
			Pharmacodynamics	Pharmacokinetics			
Omeprazole (20 mg)	Clopidogrel (75 mg)	CYP2C19*1/*1 (NM)	ADP-Ag = 45.7% ± 14.2% (p = 0.028 vs alone)	NA	Major	CYP2C19 inhibition	3,31
Omeprazole (20 mg)	Clopidogrel (75 mg)	CYP2C19*1/*1 (NM)	IPA = 51.2% (p = 0.015 vs alone)	NA	Major	CYP2C19 inhibition	3,32
Rabeprazole (20 mg)	Clopidogrel (75 mg)	CYP2C19*1/*1 (NM)	IPA = 53.5% (p = 0.035 vs alone)	NA	Substantial	CYP2C19 inhibition	3,32

Proton pump inhibitor (PPI)	Clopidogrel	CYP2C19*17 carriers (Increased)	Adjusted HR = 2.05 (1.26–23.33), p = 0.003 vs. non PPI users	NA	Major	CYP2C19 inhibition	3,33
Lansoprazole (15 mg)	Warfarin (initial dose 3 mg)	NM<br><br>IM	Incidence of hemorrhagic complications = 2 cases (4.8%)<br>Incidence = 6 cases (14.6%) (p = 0.0172 vs NM and PM)	NA	Moderate<br><br>Substantial	CYP2C19 inhibition	3,34
Omeprazole (20 mg)	R-warfarin (10 mg)	CYP2C19*1/*1 (NM)	PT-INR max = 1.62 (1.42–41.82). p = 0.252 vs. before	AUC ↑ 20%	Substantial	CYP2C19 inhibition	3,35
Omeprazole (20 mg)	S-warfarin (10 mg)	CYP2C19*1/*1 (NM)	NA	AUC ↑ 7%	Moderate	CYP2C19 inhibition	3,35
Ticlopidine (200 mg)	Omeprazole (20 mg)	CYP2C19*1/*1 (NM)<br>CYP2C19*1/*2; *1/*3 (IM)	NA	AUC ↑ 522%<br>AUC ↑ 401%	Major<br>Major	CYP2C19 inhibition	3,36
Moclobemide (300 mg)	Omeprazole (40 mg)	CYP2C19*1/*1 (NM)	NA	AUC ↑ 107%	Substantial	CYP2C19 inhibition	3,37
Fluvoxamine (50 mg)	Omeprazole (60 mg)	CYP2C19*1/*1 (NM)<br>CYP2C19*1/*2; *1/*3 (IM)	NA	AUC ↑ 462%<br>AUC ↑ 138%	Major<br>Substantial	CYP2C19 inhibition	3,38
Fluvoxamine (50 mg)	Lansoprazole (60 mg)	CYP2C19*1/*1 (NM)<br>CYP2C19*1/*2; *1/*3 (IM)	NA	AUC ↑ 283%<br>AUC ↑ 150%	Major<br>Substantial	CYP2C19 inhibition	3,39
Fluvoxamine (25 mg bid)	R-lansoprazole (60 mg)	CYP2C19*1/*1 (NM)<br>CYP2C19*1/*2; *1/*3	NA	AUC ↑ 799%<br>AUC ↑ 327%<br>AUC ↑ 127%	Major<br>Major<br>Substantia	CYP2C19 inhibition	3,40

		(IM) CYP2C19*2/*2; *2/*3 (PM)					
Fluvoxamine (25 mg bid)	S-lansoprazole (60 mg)	CYP2C19*1/*1 (NM) CYP2C19*1/*2; *1/*3 (IM) CYP2C19*2/*2; *2/*3 (PM)	NA	AUC ↑ 1297%<br>AUC ↑ 521%<br>AUC ↑ 101%	Major<br>Major<br>Substantial	CYP2C19 inhibition	3,40
Fluvoxamine (50 mg)	Rabeprazole (20 mg)	CYP2C19*1/*1 (NM) CYP2C19*1 /*2; *1/*3 (IM)	NA	AUC ↑ 182%<br>AUC ↑ 68%	Substantial<br>Moderate	CYP2C19 inhibition	3,41
Oral contraceptives	Omeprazole (20 mg)	CYP2C19*1/*1 (NM)	MR = 1.21 (0.71–72.08) ($p < 0.05$ vs. without)	NA	Substantial	CYP2C19 inhibition	3,42
Clopidogrel (Day 1 = 300 mg; Day 2–5 = 75 mg)	Omeprazole (400 mg)	CYP2C19*1/*1 (NM)	NA	AUC ↑ 28%	Moderate	CYP2C19 inhibition	3,43
Omeprazole (40 mg)	Moclobemide (300 mg)	NM	NA	AUC ↑ 31%	Moderate	CYP2C19 inhibition	3,44
Omeprazole (20 mg)	Diazepam (0.1 mg/kg)	NM	NA	AUC ↑ 36%	Moderate	CYP2C19 inhibition	3,45
**P450 Isoform CYP2D6**							
**Effector drug (dose)**	**Substrate (dose)**	**Genotype/ phenotype†**	**Effect of interaction**		**Clinical impact of interaction**	**Possible mechanism**	**Level of evidence, [citation]**
			Pharmacodynamics	Pharmacokinetics			
Terbinafine (250 mg)	Dextro-methorphan (0.3 mg/kg)	CYP2D6*1/*1; *1/*2;*2/*2; *1/*4 (NM/IM)	NA	MR DMP/dextrorphan = 0.307, absolute change = 96.67, ($p < 0.05$ vs. before)	Major	CYP2D6 inhibition	3,46

Quinidine (100 mg bid)	R-Venlafaxine (18.75 mg bid)	NM	NA	oral CL ↓ 83%	Major	CYP2D6 inhibition	3,47
Quinidine (100 mg bid)	S-Venlafaxine (18.75 mg bid)	CYP2D6*1/*4; *1/*1 (NM/IM)	NA	AUC ↑ 285%	Major	CYP2D6 inhibition	3,47
Paroxetine (20 mg)	Desipramine (100 mg)	NM<br>PM	NA	Total CL ↓ 78%<br>Total CL ↓ 20%	Major<br>Moderate	CYP2D6 inhibition	3,48
Paroxetine (20 mg)	Aripiprazole (3 mg)	CYP2D6*1/*1; *1/*5; *1/*10 (NM/IM)<br>CYP2D6*5/*10; *10/*10; *10/*21 (IM)	NA	AUC ↑ 136%<br>AUC ↑ 29%	Substantial<br>Moderate	CYP2D6 inhibition	3,49
Diphenhydramine (50 mg tid)	Metoprolol (100 mg)	CYP2D6*1/*1; *1/*3; *1/*4; *1/*5 (NM/IM)	Heart rate reduced significantly	AUC ↑ 90%	Substantial	CYP2D6 inhibition	3,51
Diphenhydramine (50 mg tid)	Metoprolol (100 mg)	CYP2D6*1/*1; *1/*3;*1/*4; *1/*5 (NM/IM)	NA	Metabolic CL ↓ 61%	Substantial	CYP2D6 inhibition	3,51
Diphenhydramine (50 mg tid)	Metoprolol (100 mg)	NM	A significant effect on heart rate and systolic blood pressure response	AUC ↑ 61%	Substantial	CYP2D6 inhibition	3,52
Celecoxib (200 mg bid)	Metoprolol (50 mg)	CYP2D6*1/*1; *1/*2;*2/*2 (NM)<br>CYP2D6*2 /*4; *1/*4;*2/*5 (IM)	NA	AUC ↑ 103%]<br>AUC ↑ 36%	Substantial<br>Moderate	CYP2D6 inhibition	3,53
Diphenhydramine (50 mg bid)	Venlafaxine (18.75 mg bid)	CYP2D6*1/*4; *1/*1 (NM/IM)	NA	Oral CL ↓ 59%	Substantial	CYP2D6 inhibition	3,54
CYP2D6 inhibitors (paroxetine,	Tramadol (3 mg/kg)	CYP2D6*1/*1 (NM)<br>CYP2D6*1/*3;*1/*4;	NA	(+)ODT AUC ↓ 93%<br>(+) ODT AUC ↓ 82%	Substantial<br>Substantial	CYP2D6 inhibition	2,55

amiodarone, cimetidine, and ranitidine)		*1/*5;*1/*6;*1/*10; *1/*41;*10/*41;*3/*41; *4/*41;*6/*10;*6/*41 (NM/IM) CYP2D6*1/*4xN; *1/*41xN; *1/1*xN (UM)		(+) ODT AUC ↓ 83%	Substantial		

Effect of polymorphisms in drug-drug-gene interactions

**Effector drug (dose)**	Type of effector drug	**Substrate (dose)**	Alternative metabolic enzyme	**Genotype/ phenotype† of main metabolic enzyme**	**Effect of interaction**		**Clinical impact**	**Possible mechanism. level of evidence, [citation]**
**P450 Isoform CYP2D6**								
					Pharmacodynamics	Pharmacokinetics		
Cotrimoxazole (960 mg bid)	CYP2C9 inhibitor	Venlafaxine (112.5 mg)	CYP2C9; CYP2D6	CYP2C19*1/*2 (IM)	Severe tremor development	Serum concentration of venlafaxine and O-desmethyl venlafaxine increased by about 30%	Substantial	Reduced capacity of CYP2C19; CYP2C9 inhibition,2, [56]
**P450 Isoform CYP2C19**								
					Pharmacodynamics	Pharmacokinetics		
Pantoprazole (20 mg)	CYP2C19/ 3A4/ 3A5 inhibitor	Atorvastatin (20 mg)	CYP3A4	CYP2C19*2/*2 (PM)	Myopathy/ rhabdomyolysis	NA	Major	Inactive CYP2C19; CYP3A

	and substrate							inhibition,1, [57].
Clarithromycin (400 mg bid)	CYP3A4 inhibitor	Omeprazole (20 mg)	CYP3A4	CYP2C19*1/*1 (NM)<br>CYP2C19*1/*2;*1/*3 (IM)<br>CYP2C19*2/*2;*3/*3 (PM)	NA	AUC ↑ 112%<br>AUC ↑ 111%<br>AUC ↑ 134%	Substantial	Inactive CYP2C19; CYP3A inhibition,3, [58]
Clarithromycin (400 mg bid)	CYP3A4 inhibitor	R-Lansoprazole (60 mg)	CYP3A4	CYP2C19*1/*1 (NM)<br>CYP2C19*1/*2;*1/*3 (IM)<br>CYP2C19*2/*2;*2/*3 (PM)	NA	AUC ↑ 95%<br>AUC ↑ 71%<br>AUC ↑ 133%	Substantial<br>Moderate<br>Substantial	Inactive CYP2C19; CYP3A inhibition.3, [59]
Clarithromycin (400 mg bid)	CYP3A4 inhibitor	S-Lansoprazole (60 mg)	CYP3A4	CYP2C19*1/*1 (NM)<br>CYP2C19*1/*2;*1/*3 (IM)<br>CYP2C19*2/*2;*2/*3 (PM)	NA	AUC ↑ 127%<br>AUC ↑ 104%<br>AUC ↑ 113%	Substantial	Inactive CYP2C19; CYP3A inhibition, 3,[60]
Clarithromycin (400 mg bid)	CYP3A4 inhibitor	Lansoprazole (60 mg)	CYP3A4	CYP2C19*1/*1 (NM)<br>CYP2C19*1/*2;*1/*3 (IM)<br>CYP2C19*2/*2;*2/*3 (PM)	NA	AUC ↑ 62%<br>AUC ↑ 71%<br>AUC ↑ 89%	Moderate<br>Moderate<br>Substantial	Inactive CYP2C19; CYP3A inhibition,3, [60]
Fluvoxamine (25 mg bid)	CYP2C9/ 2C8/ 1A2/	Lansoprazole (60 mg)	CYP3A4	CYP2C19*1/*1 (NM)<br>CYP2C19*1/*2; *1/*3 (IM)	NA	AUC ↑ 283%<br>AUC ↑ 150%	Major<br>Substantial	Inactive CYP2C19;

	2C19/ 3A4 inhibitor							CYP3A inhibition,3, [61]
Clarithromycin (500 mg bid)	CYP3A4 inhibitor	Esomeprazole (40 mg)	CYP3A4	CYP2C19*1/*1 (NM)<br>CYP2C19*2 /*2; *2/*3 (PM)	NA	AUC ↑70%<br>AUC ↑114%	Moderate<br>Substantial	Inactive CYP2C19; CYP3A inhibition,3,[61]
Ketoconazole (100 mg)	CYP3A4 inhibitor	Omeprazole (20 mg)	CYP3A4	NM<br>PM	NA	AUC ↑37%<br>AUC ↑95%	Moderate<br>Substantial	Inactive CYP2C19; CYP3A inhibition,4,[62]
**P450 Isoform CYP2D6**								
Clarithromycin and paroxetine	CYP3A4 inhibitor and CYP2D6 inhibitor	Venlafaxine (75 mg)	CYP3A4	CYP2D6*1/*1; *1/*2 (NM)<br>CYP2D6*1/*1; *1/*2 (NM)<br>CYP2D6*10/*10 (IM)<br>CYP2D6*10/*10 (IM)	NA	After clarithromycin: AUC ↑8%<br>After clarithromycin and paroxetine: AUC ↑328%<br>After clarithromycin: AUC ↑31%<br>After clarithromycin and paroxetine: AUC ↑124%	Minimal<br>Moderate<br>Major<br>Substantial	Inactive CYP2D6; CYP3A inhibition,3, [63]
Clarithromycin	CYP3A4 inhibitor	Hydrocodone	CYP3A4 CYP2D6*41 (decreased)	Fatal opioid toxicity		Blood concentration of hydrocodone = 0.14 μg/ml (toxic level)	Major	Inactive CYP2D6; CYP3A inhibition,1, [64]

Clarithromycin and voriconazole	CYP3A4 inhibitor	Codeine (25 mg tid)	CYP3A4	UM	Coma and respiratory depression	The blood level of morphine was 80 μg/l (expected range = 1–4 μg/l)	Major	Increase CYP2D6 activity and CYP3A4 inhibition; acute renal failure,2, [65]
Itraconazole (200 mg bid)	CYP3A4 inhibitor	Haloperidol (5 mg)	CYP3A4	CYP2D6*1/*1 (NM) CYP2D6*10/*10 (IM)	BARS = 0.86 ± 1.46 ($p > 0.05$ vs placebo) BARS = 1.86 ± 1.77 ($p < 0.05$ vs CYP2D6*1/*1) AUC ↑ 99%	AUC ↑ 55%	Substantial Major	Inactive CYP2D6; CYP3A inhibition,3, [66]
Itraconazole (200 mg)	CYP3A4 inhibitor	Risperidone (2 to 8 mg)	CYP3A4	CYP2D6*1/ *1; *1/ *10; *10/ *10 (NM/IM)	Scores on Brief Psychiatric Rating Scale decreased significantly but the scores on the Udvalg For Kliniske Undersogelser Side Effect Rating Scale were not changed	Dose normalized plasma concentration ↑ 69%	Substantial	Inactive CYP2D6; CYP3A inhibition,4, [67]
Ketoconazole (200 mg bid)	CYP3A4 inhibitor	Fesoterodine (8 mg)	CYP3A4	NM	NA	AUC ↑ 131%	Substantial	Inactive CYP2D6; CYP3A inhibition,3, [68]
Rifampicin (600 mg)	CYP3A4 inducer	Fesoterodine (8 mg)	CYP3A4	NM	NA	AUC ↓ 77%	Substantial	Inactive CYP2D6;

								CYP3A4 induction,3,[69]

Source: [24] Bahar MA, Setiawan D, Hak E, Wilffert B. Pharmacogenetics of drug–drug interaction and drug–drug–gene interaction: a systematic review on CYP2C9, CYP2C19 and CYP2D6. Pharmacogenomics 2017 18(7), 701–739.

Notes: Only medications included likely to be prescribed by primary care physicians, those prescribed by specialists excluded.

ADP-AG (adenosine diphosphate-induced platelet aggregation); AUC (Area under curve); BARS (Barnes Akathisia Rating Scale); Cl (clearance); bid (twice daily); IM (intermediate metabolizer); MR (metabolic ratio); NA (not available); NM (normal metabolizer); PM (poor metabolizer);ODT (O-desmethyl tramadol); PT-INR (Prothrombin time expressed as International Normalized Ratio); tid (three times daily); UM (ultra metabolizer)

Strength of evidence 0-4 based on criteria (in Supplementary Data to article by Bahar et al.)
